# 

**Published:** 1878-01

**Authors:** 


**Subscription Blank Enclosed.**

**Vol. XIII.** **JANUARY, 1878.** **No. 4.**

SURG [illegible] OF[illegible]

### THE BISTOURY:

### A QUARTERLY JOURNAL,

*Devoted to the Exposition of Charlatanism in Medicine and the Education of the People upon Medical Subjects!*

**Thad S. Up de Graff, M. D., Editor.**

*Terms of Subscription, Fifty Cents per Annum, in Advance.*

ELMIRA, N. Y.

PRINTED FOR THE PROPRIETOR, AT THE DAILY ADVERTISER JOB PRINTING HOUSE.

